# Supplementary material for: The interactome of intact mitochondria by cross-linking mass spectrometry provides evidence for coexisting respiratory supercomplexes
Source: Mol Cell Proteomics. 2017 Dec 8;17(2):216–32. doi: 10.1074/mcp.RA117.000470 (PMC5795388; doi:10.1074/mcp.RA117.000470)
Supplement: Supplemental Data [file supp_RA117.000470_133922_0_supp_23978_fzffwf.zip › spectra_annotation/mito_DR_spectra_annotation/356-1-6-1-6-1.pdf]

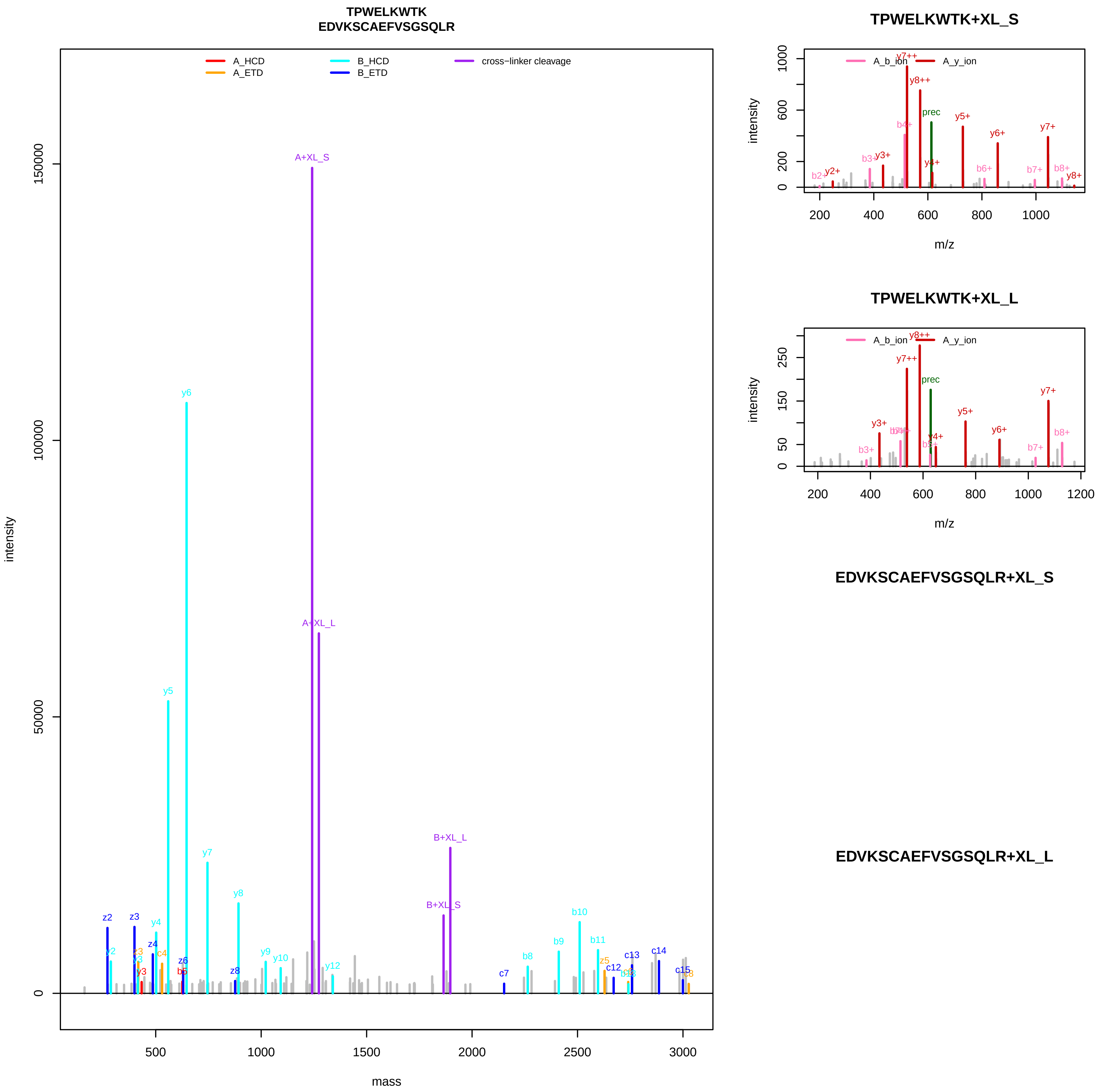
TPWELKWTK
EDVKSCAEFVSGSQLR
A_HCD
A_ETD
B_HCD
B_ETD
cross−linker cleavage
intensity
mass
A+XL_S
A+XL_L
B+XL_L
B+XL_S
y6
y5
y7
y8
y4
y2
y9
y10
y12
z2
z3
z4
z6
z8
z5
c4
c7
c12
c13
c14
c15
b8
b9
b10
b11
TPWELKWTK+XL_S
TPWELKWTK+XL_L
A_b_ion
A_y_ion
prec
y7++
y8++
y5+
y6+
y7+
y3+
y4+
y2+
y8+
b2+
b3+
b4+
b6+
b7+
b8+
m/z
EDVKSCAEFVSGSQLR+XL_S
EDVKSCAEFVSGSQLR+XL_L
